# Artri King-induced Hypothalamic-pituitary-adrenal Axis Disruption: A Report of 3 Cases

**DOI:** 10.1210/jcemcr/luad154

**Published:** 2023-12-19

**Authors:** Elyssa Ann Berg, Lisa Dao, Run Yu, Carolina Hurtado

**Affiliations:** Department of Internal Medicine, UCLA, Los Angeles, CA 90095, USA; Department of Endocrinology, UCLA, Los Angeles, CA 90095, USA; Department of Endocrinology, UCLA, Los Angeles, CA 90095, USA; Department of Internal Medicine, Division of Endocrinology, Los Angeles General Medical Center, Los Angeles, CA, USA

**Keywords:** cushing syndrome, supplement use, Artri King, iatrogenic adrenal insufficiency

## Abstract

We present a case series of 3 patients who developed iatrogenic hypothalamic-pituitary-adrenal axis disruption while taking Artri King, an over-the-counter supplement marketed for joint pain that is reported to contain dexamethasone not listed on the supplement's label. Patient 1, a 58-year-old woman, presented with persistent hyponatremia, weight gain, proximal muscle weakness, dorsocervical fat pad, and new, red striae on her breast and abdomen in the setting of Artri King use. Her dexamethasone level was elevated (Table 1), confirming the suspicion of dexamethasone content in this supplement. Patient 2, a 55-year old woman, had presented with cushingoid features and a low morning cortisol level (Table 1) in the setting of Artri King use. Patient 3, a 59-year-old man, presented with poorly controlled diabetes in the setting of Artri King use and an elevated serum dexamethasone level. Supplements containing hidden glucocorticoids can cause not only iatrogenic Cushing syndrome, but also adrenal suppression, providing a diagnostic challenge for providers.

## Introduction

Cushing syndrome is a hormonal disorder that occurs when the body is exposed to high glucocorticoid levels over an extended period. This can cause a wide range of symptoms, including weight gain, elevated blood pressure, and muscle weakness. Cushing syndrome can be caused by the overproduction of cortisol by the adrenal glands or by the prolonged use of exogenous glucocorticoids, which are synthetic versions of cortisol often used to treat certain inflammatory conditions (1). However, there is growing evidence that certain supplements that contain undisclosed glucocorticoids can potentially cause iatrogenic Cushing syndrome (2, 3). More recently, 1 of these over-the-counter supplements, known as Artri King, advertised to treat arthritis, has been implicated as a cause of iatrogenic Cushing syndrome and concomitant adrenal insufficiency because of its dexamethasone content not listed on its label (4). Here, we present 3 cases in which patients unknowingly consumed glucocorticoids through use of the Artri King supplement and subsequently developed iatrogenic Cushing syndrome or hypothalamic-pituitary-adrenal (HPA) axis disruption.

## Case Presentation

### Case 1

**Figure 1.**
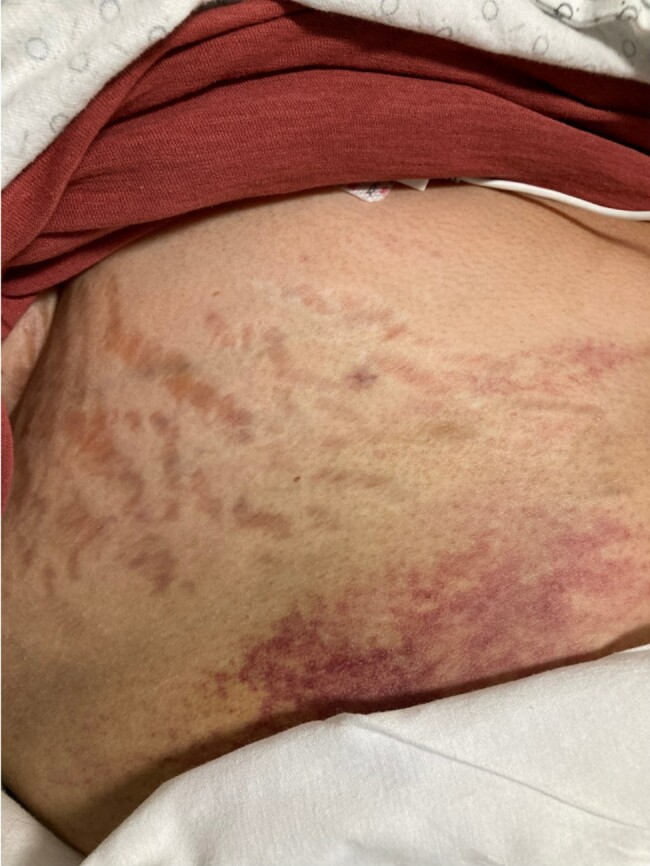
Striae on the right breast seen in patient 1.

**Figure 2.**
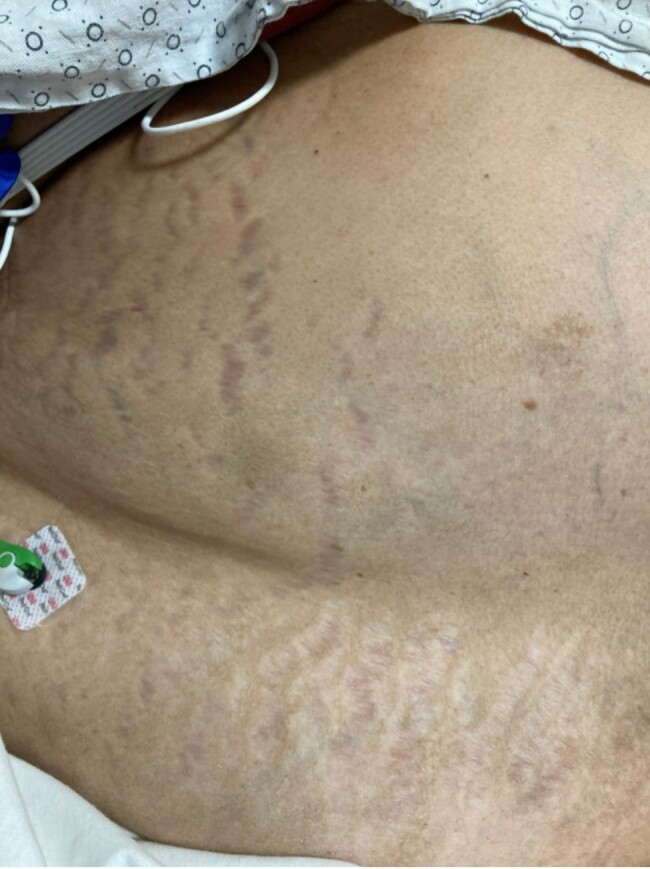
Striae on the abdomen seen in patient 1.

**Figure 3.**
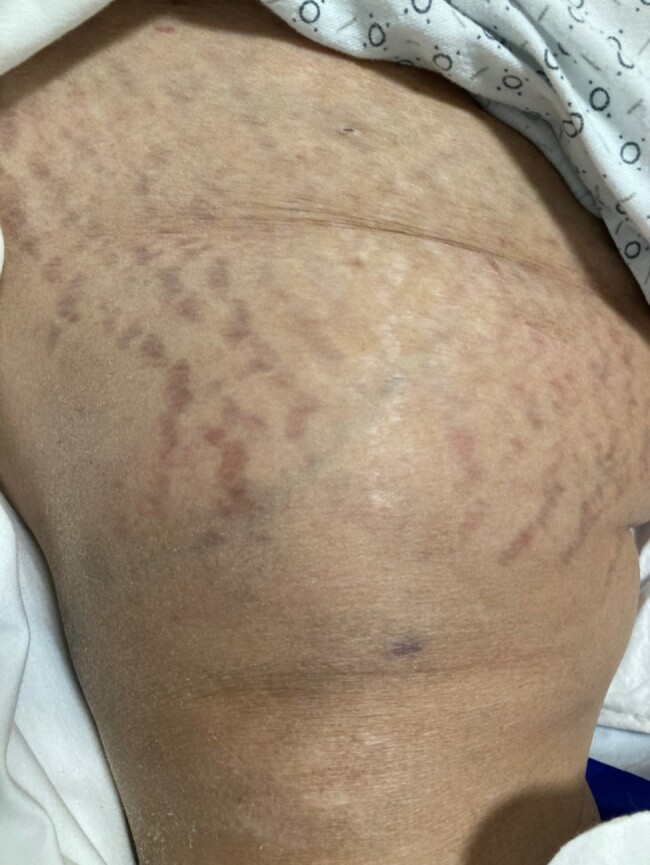
Striae on the right thigh seen in patient 1.

A 58-year-old Hispanic woman presented to the hospital with persistent hyponatremia. During her initial admission, she was diagnosed with adrenal insufficiency (AI), which was attributed as the cause of her hyponatremia. She was prescribed hydrocortisone but self-discontinued this on discharge. On repeat presentation 3 months later because of recurrent hyponatremia, it was noted that she had physical examination findings of Cushing syndrome, including increased weight gain, proximal muscle weakness, a prominent dorsocervical fat pad, and wide, red striae on the breast (Fig. 1), abdomen (Fig. 2), and thighs (Fig. 3). These new findings had developed over the course of several months. On further review of her medications, it was noted that she was taking Artri King, a supplement she attributed to significantly helping her arthritis symptoms. She endorsed taking 3 tablets per day for the past 6 months to 1 year before presentation and was not aware of it containing any glucocorticoids.

### Case 2

A 55-year-old Hispanic woman presented to endocrinology clinic for type 2 diabetes, worsening hypertension (blood pressure readings elevated to 179/81), and weight gain of 40 pounds over a 4-month period (body mass index, 45.31 kg/m^2^). Her other complaints included facial rounding and alopecia areata.

### Case 3

A 59-year-old Hispanic man was being evaluated in endocrinology clinic for uncontrolled type 2 diabetes. Over the course of his visits, his glycemic control worsened and included increasing insulin requirements. Despite not having any physical examination features of Cushing syndrome, his worsening glycemic control led to evaluation of hypercortisolism.

## Diagnostic Assessment

### Case 1

On her initial admission, this patient’s workup revealed a low morning cortisol and ACTH (Table 1). A cosyntropin stimulation test was performed with 1-hour peak cortisol of 10 mcg/dL (275.9 nmol/L), which confirmed the diagnosis of adrenal insufficiency. Additional pituitary laboratory results were notable for low LH and FSH with normal prolactin and thyroid function tests. Because of the laboratory results suggesting secondary AI and inappropriately low gonadotropins in a postmenopausal woman, a pituitary magnetic resonance imaging scan was ordered to evaluate for a possible pituitary abnormality but was unable to be completed because of the patient's body habitus (body mass index, 54.47 kg/m^2^). On discovery of Artri King use, a dexamethasone level was obtained and noted to be elevated (Table 1) despite the patient not receiving any other source of dexamethasone. She was subsequently diagnosed with adrenal insufficiency secondary to iatrogenic glucocorticoid use from Artri King.

**Table 1.** Laboratory results for three patient cases

Laboratory value	Patient 1	Patient 2	Patient 3
Dexamethasone (RR <20 ng/dL, < 509.6 pmol/L)	164 ng/dL (4178.7 pmol/L)		116.1 ng/dL (2958.2 pmol/L)
Cortisol (morning draw) (RR 8-25 mcg/dL, 220.7-689.8 nmol/L)	1 mcg/dL (27.6 nmol/L)	1.1 mcg/dL (30.4 nmol/L)	0.6 mcg/dL (16 nmol/L)
ACTH (RR 4-48 pg/mL, 0.9-10.6 pmol/L)	<2 pg/mL (<0.4 pmol/L)		<2 pg/mL (<0.4 pmol/L)
Cortisol after cosyntropin (RR >14-18 mcg/dL, 386-500 nmol/L)	10 mcg/dL (275.9 nmol/L)		
Cortisol after dexamethasone suppression test (RR <1.8 mcg/dL, < 50 nmol/L)		0.6 mcg/dL (16.6 nmol/L)	
Late night salivary cortisol (RR 0.01-0.09 µg/dL, 0.3-2.5 nmol/L),		0.01 µg/dL (0.3 nmol/L), < 0.01 µg/dL (<0.3 nmol/L)	

Abbreviation: RR, reference range.

All results and reference ranges are reported in conventional units and Système International (SI) unit, which are reported in parenthesis.

### Case 2

Adrenal magnetic resonance imaging previously performed showed no evidence of adrenal adenoma. Because of multiple symptoms that were concerning for Cushing syndrome, she completed a 1-mg dexamethasone suppression test and 2 late night salivary cortisol tests, all which were normal and suggested against endogenous hypercortisolism. Furthermore, her morning serum cortisol levels were low, findings more consistent with AI. This led to suspicion for medication-induced iatrogenic Cushing syndrome and secondary AI. Further evaluation of her medication list led to the discovery that she was taking Artri King for arthritis pain.

### Case 3

Similar to the other cases, serum cortisol and ACTH levels were low (Table 1). Medication side effects were suspected as the cause of cortisol and ACTH suppression, and a review of this patient’s prescriptions and supplements revealed the use of Artri King to help with arthritis-related pain for approximately 2 weeks. He denied use of any other exogenous glucocorticoid or supplements. Despite this, his serum dexamethasone level was elevated (Table 1), confirming the suspicion that the patient was unknowingly taking dexamethasone from the Artri King supplements.

## Treatment

### Case 1

The patient was advised to discontinue this supplement and started on physiologic glucocorticoid replacement instead with hydrocortisone.

### Case 2

The patient was instructed to discontinue Artri King and start physiologic glucocorticoid replacement with hydrocortisone 15 mg every morning and 5 mg in the afternoon.

### Case 3

The patient was advised to discontinue this supplement.

## Outcome and Follow-up

### Case 1

Lost to follow-up.

### Case 2

She eventually was able to taper off all glucocorticoids and had recovery of her HPA axis after 4 months. Cessation of the supplement led to dramatic improvement in her weight and other cushingoid symptoms.

### Case 3

On discontinuation of Artri King, the patient did not develop any symptoms concerning for secondary AI and glucocorticoid replacement was not required. His glycemic control improved after stopping the supplement.

## Discussion

Artri King, a supplement marketed for joint pain and arthritis, has come under scrutiny because of the presence of undisclosed glucocorticoids. In its label, it lists ingredients such as chondroitin, glucosamine, turmeric, vitamin C, omega 3, and collagen, among others. In April 2022, the US Food and Drug Administration released a public notification advising consumers not to use or purchase Artri King after its laboratory analysis confirmed it contained diclofenac and dexamethasone not listed on the product's label (4). In the cases we have presented, the most likely cause of cushingoid features was due to sustained usage of the Artri King supplement purchased outside of the United States. There were no other identifiable sources of exogenous glucocorticoids, including topical, inhaled, or intranasal preparations. Our findings align with several other documented cases of Artri King-associated Cushing syndrome, emphasizing the need for increased awareness among health care professionals and the general public (3, 5). The importance of addressing this matter cannot be overstated because of the potential health risks associated with the use of hidden glucocorticoids. Chronic use of Artri King or similar products containing undisclosed glucocorticoids can lead to iatrogenic Cushing syndrome and secondary AI by negative feedback on the HPA axis and even cause life-threatening adrenal crisis. Patients who rely on these supplements may unknowingly expose themselves to the adverse effects of long-term glucocorticoid use without proper medical supervision. One significant issue is that patients may not report supplements as active medications and the diagnosis may be delayed. Thus, any patient with clinical signs of Cushing syndrome, low cortisol or ACTH levels, and no other obvious source of exogenous glucocorticoid use should have a synthetic glucocorticoid screening test completed. Increased awareness and understanding of the presence of hidden exogenous glucocorticoids in dietary supplements are vital to protect the health and well-being of individuals who use these products. Health care professionals, including endocrinologists, primary care physicians, and pharmacists, play a crucial role in educating patients about the potential risks associated with unregulated supplements.

## Learning Points

- Herbal supplements can contain hidden ingredients, such as glucocorticoids.
- Artri King is a supplement used for the treatment of arthritis known to contain dexamethasone not listed on the product's label.
- Use of these supplements can not only lead to iatrogenic Cushing syndrome, but also secondary adrenal insufficiency.
- Health care providers should always get a thorough drug and supplement history when they suspect Cushing syndrome or secondary adrenal insufficiency. Any patient that presents with inexplicably subnormal cortisol or ACTH levels should have a synthetic glucocorticoid screen performed.

## Data Availability

Data sharing is not applicable to this article as no datasets were generated or analyzed during the current study.

## Abbreviations

AI: adrenal insufficiency
HPA: hypothalamic-pituitary-adrenal

## References

[1] Newell-Price J, Bertagna X, Grossman AB, Nieman LK. Cushing's syndrome. Lancet. 2006;367(9522):1605‐1617. 16698415 10.1016/S0140-6736(06)68699-6

[2] Chong Y, Ching C, Ng S, Mak TW. Corticosteroid adulteration in proprietary Chinese medicines: a recurring problem. Hong Kong Med J. 2015;21(5):411‐416. 26314568 10.12809/hkmj154542

[3] Patel R, Sherf S, Lai NB, Yu R. Exogenous cushing syndrome caused by a “herbal” supplement. AACE Clin Case Rep. 2022;8(6):239‐242. 36447831 10.1016/j.aace.2022.08.001 PMC9701910

[4] US Food and Drug Administration . Public Notification: Artri King Contains Hidden Drug Ingredients. FDA; 2022.

[5] Dunn C, Amaya J, Green P. A case of iatrogenic Cushing's Syndrome following use of an over-the-counter arthritis supplement. Case Rep Endocrinol. 2023;2023:1‐3. 10.1155/2023/4769258 PMC10024620 36941974

